# Recognition of Consumer Preference by Analysis and Classification EEG Signals

**DOI:** 10.3389/fnhum.2020.604639

**Published:** 2021-01-13

**Authors:** Mashael Aldayel, Mourad Ykhlef, Abeer Al-Nafjan

**Affiliations:** ^1^Information Technology Department, College of Computer and Information Sciences, King Saud University, Riyadh, Saudi Arabia; ^2^Information System Department, College of Computer and Information Sciences, King Saud University, Riyadh, Saudi Arabia; ^3^Computer Science Department, College of Computer and Information Sciences, Imam Muhammad ibn Saud Islamic University, Riyadh, Saudi Arabia

**Keywords:** deep learning, feature extraction, customer neuroscience, classification, signal processing, neuromarketing

## Abstract

Neuromarketing has gained attention to bridge the gap between conventional marketing studies and electroencephalography (EEG)-based brain-computer interface (BCI) research. It determines what customers actually want through preference prediction. The performance of EEG-based preference detection systems depends on a suitable selection of feature extraction techniques and machine learning algorithms. In this study, We examined preference detection of neuromarketing dataset using different feature combinations of EEG indices and different algorithms for feature extraction and classification. For EEG feature extraction, we employed discrete wavelet transform (DWT) and power spectral density (PSD), which were utilized to measure the EEG-based preference indices that enhance the accuracy of preference detection. Moreover, we compared deep learning with other traditional classifiers, such as k-nearest neighbor (KNN), support vector machine (SVM), and random forest (RF). We also studied the effect of preference indicators on the performance of classification algorithms. Through rigorous offline analysis, we investigated the computational intelligence for preference detection and classification. The performance of the proposed deep neural network (DNN) outperforms KNN and SVM in accuracy, precision, and recall; however, RF achieved results similar to those of the DNN for the same dataset.

## 1. Introduction

Neuromarketing or consumer neuroscience is an emerging disciplinary area that connects the affective and cognitive aspects of customer behavior utilizing neuroimaging tools such as brain-computer interfaces (BCIs). BCIs play the role of a communication tool between humans and computer systems without any external devices or muscle intervention to issue commands, control, or complete an interaction. BCI research and development initially considered as an assistive technology aimed to help individuals with physical disabilities in various aspects such as communication, control, and mobility. In recent times, alternative BCI applications for healthy humans have been developed, and an increasing number of these re-searches target fields such as neuromarketing (Al-Nafjan et al., 2017a). Electroencephalography (EEG) is a practical, versatile, affordable, portable, and non-invasive technique for performing repetitive sessions, tasks, and observations. EEG-based BCIs have gained increasing interest in the literature from various scientific disciplines (Al-Nafjan et al., 2017a).

In neuromarketing, EEG-based preference detection seeks to provide insights into an individual's experience with a variety of products and media as well as his responses to market stimuli. It is a well-known fact that consumer emotions impact decision-making. On the other hand, consumer's emotions can strongly be influenced by many internal and external factors. The detection and recognition of a consumer's emotional state thus reveal true consumer preferences (Aldayel et al., 2020). Although several studies have been conducted on EEG-based emotion recognition (Ramadan et al., 2015), EEG-based studies for detecting preferences in consumers are in a very early phase. Furthermore, only a few preference-recognition studies have evaluated passive BCIs compared to the number of active BCIs. Additional research that employee BCIs to assess unconscious customer preferences is therefore needed, as opposed to research on BCIs for direct control actions (van Erp et al., 2012).

An EEG-based preferences detection system helps us understanding consumer preferences and behavior to understand how one makes a buying decision. It will help marketers and organizations acting upon them to increase customer satisfaction, positive customer experiences, consumer loyalty, and revenue. (Aldayel et al., 2020).

Although the neuromarketing field has evolved significantly in the last decade; it still has not been fully implemented in the separated academic fields in marketing research. This is because marketing researchers lack training on systematic cognitive practices in neuroscience. In addition, marketing researchers have previously doubted the implications of violating ethical rules and the privacy of consumers when using neuroscience technologies for commercial purposes. However, there are still reservations against the use of neuromarketing to extract specific knowledge of customers (Ait Hammou et al., 2013). Consequently, the potential use of EEG data during passive observations to derive product preferences remains an open debate (Telpaz et al., 2015). Accordingly, only a few neuromarketing research on advertising efficiency (Morin, 2011) were reported. This research aims to thoroughly examine the preference detection in neuromarketing using EEG indices. We chose these EEG indices based on an analysis of neural correlations of the preference that was explained in our previous research (Aldayel et al., 2020). We employed two approaches for the extraction of EEG features, namely, discrete wavelet transform (DWT) and power spectral density (PSD).

These approaches were used to measure the EEG-based preference indices. The preference indices enhance the accuracy of preference prediction. In fact, to the best of our knowledge, this is the first study that examines in detail the effect of preference indicators in enhancing the performance of classification algorithms. Furthermore, we analyzed the performance of deep learning with other conventional classification algorithms, such as k-nearest neighbor (KNN), random forest (RF), and support vector machine (SVM).

The remainder of this paper is arranged as follows: section 2 introduces the main concepts of this study with background details; section 3 presents the related works; section 4 describes the research methodology, i.e., the experiments with EEG data; section 5 discusses the evaluation results; and, finally, section 6 presents the conclusion.

## 2. Background

In this section, we provide an overview of BCI-based preference detection and examine EEG-based preference indices.

### 2.1. BCI-Based Preference Detection

**Figure 1:**
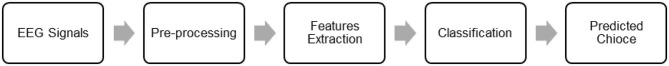
EEG-based BCI for preference recognition.

This section explains the design process of neuromarketing experiments for anticipating customer preferences and choices. First, a customer places a BCI device on his/her head. Then, the customer looks at the products while EEG data are recorded at the same time on the BCI. Next, the customer rates his/her preference on each product using a nine-point subjective ranking scale. After viewing all products, the subjective ranks need to be manually labeled as “preferred” or “unpreferred.” Next, the recorded EEG signals go through preprocessing and feature extraction. The training and prediction of the classifier are based on the consumer's choice (subjective ranks). The proposed BCI system for preference detection is shown in Figure 1. This system has three fundamental modules: signal preprocessing, feature extraction, and classification modules.

### 2.2. EEG-Based Preference Indices

This section explains the preference indicators based on EEG signals. Based on our literature review (Aldayel et al., 2020), we defined the following four EEG indices to measure people's responses to marketing stimuli: the approach-withdrawal (AW) index, valence, choice index, and effort index. Such indices help marketers in realizing the reactions of consumers to products (Cartocci et al., 2017; Cherubino, 2018).

#### 2.2.1. AW Index

The AW index measures the frontal alpha asymmetry reflected the difference between the left and right hemispheres; that is, the percentage of participation of the left hemisphere compared to the right one in the frontal alpha band (Cartocci et al., 2017; Touchette and Lee, 2017; Cherubino, 2018; Ramsøy et al., 2018). Several studies have shown the efficacy and precision of frontal alpha asymmetry as an essential determinant in emotion and neuromarketing research (Cartocci et al., 2017; Touchette and Lee, 2017; Al-Nafjan et al., 2017b; Cherubino, 2018; Modica et al., 2018; Ramsøy et al., 2018).

#### 2.2.2. Effort Index

This measure is described as the activity level of the frontal theta in the prefrontal cortex. Higher theta activity has been associated with higher levels of task difficulty and complexity in the frontal area. It is an indication of cognitive processing arising from mental exhaustion (Modica et al., 2018) and has been frequently studied in neuromarketing research (Vecchiato et al., 2010, 2011; Boksem and Smidts, 2015; Telpaz et al., 2015; Modica et al., 2018). This reveals the significance of handling emotional changes for the formation of sustainable memory in commercials (Cartocci et al., 2017).

#### 2.2.3. Choice Index

The choice index measures the frontal irregular fluctuations in beta and gamma, frequently associated with the actual stage of decision-making. It has been the most associated marker of willingness to pay for assessing customer desire and preferences, particularly in the gamma band. Higher gamma and beta implied greater neural activity of the left frontal area, while smaller amounts are associated with greater neural activity of the right area (Ramsøy et al., 2018).

#### 2.2.4. Valence

Asymmetrical activation of the frontal hemisphere was correlated to preferences interpreted as valence, that is, the orientation of affective status of a consumer). Activation of the right and left prefrontal area is related to negative and positive values of valence, respectively. A large number of studies support the theory that frontal EEG asymmetry can be a measure of valence (Al-Nafjan et al., 2017b).

## 3. Related Work

EEG-based preference classification normally includes the spectral conversion of waveforms into features exploited by data-mining algorithms, which are trained on labeled data to forecast whether preferences are presently being detected. The preference classification of EEG varies from binary labels such as (“like” vs. “dislike”) and (most favored vs. least favored) to multiple ordinal labels in the form of ranks, such as the nine-scale rank or five-scale rank. Several preference studies have used more than two algorithms of classification to find tuned classifiers for a set of features (Hwang et al., 2013). Chew et al. (Ramadan et al., 2015) evaluated user preferences of aesthetics displayed as virtual three-dimensional objects. The frequency bands were used as features for the EEG classification into two classes—“like” and “dislike”—using SVM and KNN and achieving an accuracy of 75 and 80%, respectively. These results, however, are not considered credible since the authors used a relatively low dataset of five subjects. In their extended research (Teo et al., 2017, 2018b), the authors raised the number of subjects to 16 but did not obtain better results.

By integrating EEG measures with questionnaire measures, Hakim et al. (2018) obtained an accuracy of 68.5% using the SVM to determine the most and least preferred items. Combining classifiers, such as boosting, voting, or stacking, can be used to gather multiple classification algorithms by integrating their outcomes and/or training them to complement each other and improve their performance (Lotte et al., 2018). The choice of classifiers in a BCI system is mainly dependent on both the type of mental signals acquired and the setting in which the application is used. LDA and SVM, however, are the most widely used classification algorithms and were used in over half of the EEG-based BCI experiments. Some works employed graph-based deep learning to study attention behavior (Zhang et al., 2019, 2020). Table 1 summarizes several studies in neuromarketing in which various classifiers were used to achieve the most accurate accuracy in predicting customer preferences.

**Table 1:** Classification algorithms employed for preferences detection in neuromarketing.

**References**	**Classification Algorithm**	**Class**	**Best accuracy (%)**
Chew et al., 2016	SVM	1. Liked 2. Disliked	75
	KNN		80
Kim et al., 2015	SVM	1. Preferred image 2. Unnoticed image	83.64
Hadjidimitriou and Hadjileontiadis, 2012, 2013	KNN	1. Liked 2. Disliked	91.02
Pan et al., 2013	SVM	1. Liked 2. Disliked	74.77
Moon et al., 2013	Quadratic discriminant analysis	1. Most preferred 2. Preferred 3. Less preferred 4. Least preferred	97.39
	KNN		97.99
Teo et al., 2017, 2018a,b	DNN	1. Liked 2. Disliked	74.38
	SVM		60.19
Hakim et al., 2018	Logistic Regression	1. Most favored 2. Least favored	67.32
	SVM		68.50
	KNN		59.98
	Decision trees		63.34
Yadava et al., 2017	DNN	1. Liked 2. Disliked	60.10
	SVM		62.85
	RF		68.41
	HMM		70.33

Our review in Aldayel et al. (2020) highlighted the need to use further features and fusion of classifiers to boost the accuracy of the prediction. In this study, we used a publicly available neuromarketing dataset (Yadava et al., 2017) that was previously used (Yadava et al., 2017) in building a predictive model for consumer product choice from EEG data. By using a passive BCI, researchers studied the influences of gender and age on consumer preferences in terms of like/dislike. However, all indices of EEG-based preference recognition have not been combined in any study. To the best of our knowledge, this is the first in-depth investigation of the effect of preference indicators in enhancing the performance of classification algorithms.

## 4. Materials and Methods

The outcome of preference detection is dependent on the choices of algorithms for feature extraction and classification. In this study, we examined the probability that two affective levels, namely, “like” and “dislike,” could be identified employing different feature combinations of EEG indices as well as different approaches of feature extraction and classification algorithms. We chose these EEG indices based on an analysis of neural correlations of the preference that was explained in our previous research (Aldayel et al., 2020). For EEG feature extraction, we used DWT and PSD. Then, the PSD features were used to calculate the EEG-based preference indices. We applied deep learning classification to identify approaches of using intelligent computational modeling in the form of classification algorithms as these approaches can effectively reflect the subjects' preferred states. Moreover, we compared the efficiency of deep learning with other traditional classifiers, such as SVM, RF, and KNN. We developed our model in Python programming language using the Scikit-Learn, SciPy, and MNE and Keras packages for machine learning, EEG preprocessing and filtration, and signal processing and deep learning, respectively.

**Figure 2:**
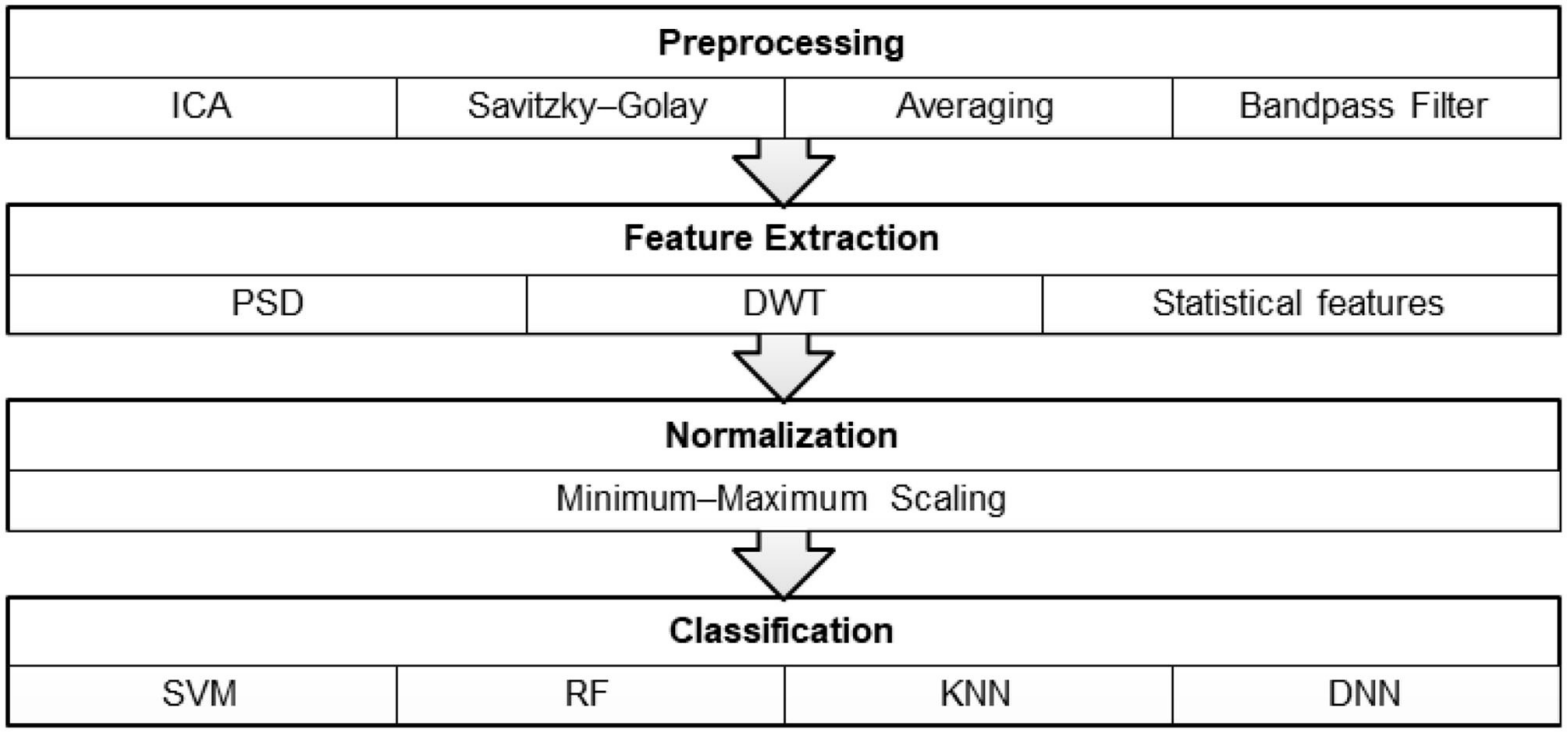
Architecture of the consumer preference prediction system.

In this section, we present our methods and describe the architecture of the proposed EEG-based preference recognition. First, we examine the neuromarketing benchmark dataset and labeling of preferences states. Then, we illustrate how to extract features from EEG signals. Lastly, we explain the DNN classifier for preference detection. Figure 2 presents the methods used in the consumer preference prediction system.

### 4.1. Dataset

This section describes a publicly available EEG dataset (Table 2) that has been used (Yadava et al., 2017) in neuromarketing experiments. The Emotiv EPOC+ headset was used to record EEG data. Twenty-five users participated, and their EEG data were recorded while they watched products on a computer screen. The age of the users ranged from 18 to 38 years. A set of 14 diverse products, each with three variations, were selected. A total of 42 (= 14 × 3) diverse product pictures were then generated, and 1050 (= 42 × 25) EEG data were therefore logged for all users. The EEG data were downsampled to 128 Hz and preprocessed to 14 channels, resulting in 25 documents or one document per user. The EEG features were collected from 14 channels placed at AF3, F7, F3, FC5, T7, P7, O1, O2, P8, T8, FC6, F4, F8, and AF4 locations. Responses in the form of either “like” or “dislike” were collected from the users for each product. Each product was presented for 4 s, and EEG data were logged simultaneously. After each image was presented, the preferred choice of the user was collected.

**Table 2:** Affective dataset description.

**Preference model**	**Binary (like-dislike)**
Stimul	Visual-based stimuli (4 s per product picture )
Participants	Twenty-five participants, aged 18–38
Trials	1,050 trials (42 trials for each subject)
EEG device	Emotiv EPOC+ device includes 14 channels
Experimental method	Each user viewed and evaluated his or her preferences toward 42 pictures of products in form of either like or dislike.

Since consumers may not be able to express their preferences when asked to clearly articulate them, their subjective labeling is not sufficient. We extracted true hidden preferences (i.e., the ground truth table) from EEG signals. We used two methods to identify preference labels (“like” or “dislike”): (1) subjective self-assessment labels collected during the experiment; and (2) valence-based labels to identify the objective preference states. In this experiment, we used different types of preference labeling to obtain more accurate results. We used the valence index as the determinant of preference to match the target preference state—“like” or “dislike.” Valence rates were categorized to lower rates if values ranged from one to five and higher rates if values ranged from six to nine. A lower valence rate is an indicator of a “dislike” preference state, while a higher valence rate is an indicator of a “like” preference state.

We used Cohen's kappa to test the agreement level between two types of labeling, namely, subjective self-assessment and valence-based labels determined from EEG. The kappa score was 0.03, which can be interpreted as a slight agreement between these labels. We also noticed there were differences in 513 of the 1050 trials, which in line with the main goal of this neuromarketing research: real and more accurate identification of preferences using EEG signals.

### 4.2. Signal Preprocessing

We first averaged the EEG signals and then resampled the frequency to 128 Hz per channel. From prior knowledge of EEG, the correlated signal frequency ranges produced by the brain during preferences states are mainly concentrated below 45 Hz. The useful frequency band in EEG signal data is therefore between 4 and 45 Hz. We used a bandpass filter ranging from 4.0 to 45.0 Hz. Subsequently, we used ICA and Savitzky–Golay filters to remove artifacts. We considered only the following electrodes in the preference calculation: AF3, F3, AF4, and F4.

### 4.3. Feature Extraction

Feature extraction aims to find important and relevant information from EEG signals. We extracted EEG frequency bands using two approaches: DWT and a PSD method named Welch. Then, we used the resulting frequency bands to calculate the preference indices. The first approach extracts a set of statistics-based features for each frequency band (details [D2-D5] and approximation [A5]) computed by DWT. The second approach stacks the features computed by PSD into a single array over the raw EEG of the channels.

#### 4.3.1. Discrete Wavelet Transform

The DWT is a time-frequency domain analysis method that decomposes signals into different coefficients. It can be defined as multi-resolution or multi-scale analysis, where each coefficient is a unique representation of mind signals. The convolution operation is a two-function multiplication process (Chen et al., 2015; Vega-Escobar et al., 2016). Each inner product results in a wavelet coefficient. Therefore, the DWT can be expressed using the following Equation (1):

(1) $$\begin{array}{l} W\left(j,k\right)=\overset{M-1}{\underset{N=0}{\sum }}f\left(n\right)\cdot \psi_{j,k}^{*}\left(n\right) \end{array}$$

where *f*(*n*) is a signal (sequence) of length *n*, and $\psi_{j,k}^{*}\left(n\right)$ is scaling wavelet function. DWT decomposition can be implemented as a group of high- and low-pass filters in a filter bank. The outputs of the low-pass filters are called approximation coefficients, and those of the high-pass filters are called wavelet detail coefficients. After the filtering, the signal is down-sampled by a factor of two based on the Nyquist Theorem, resulting in a frequency band ranging between *f*_*n*_/2 and *f*_*n*_. Assuming *f*_*s*_ sampling frequency and *L* decomposition level, every detail coefficient frequency is related to the sampling frequency rate *f*_*s*_ of the raw signals, given by *f*_*n*_ = *f*_*s*_/2*L* + 1. The number of wavelet decomposition levels and the selection of a proper wavelet technique are critical to achieving DWT analysis accuracy (Chen et al., 2015; Vega-Escobar et al., 2016; Yadava et al., 2017).

**Figure 3:**
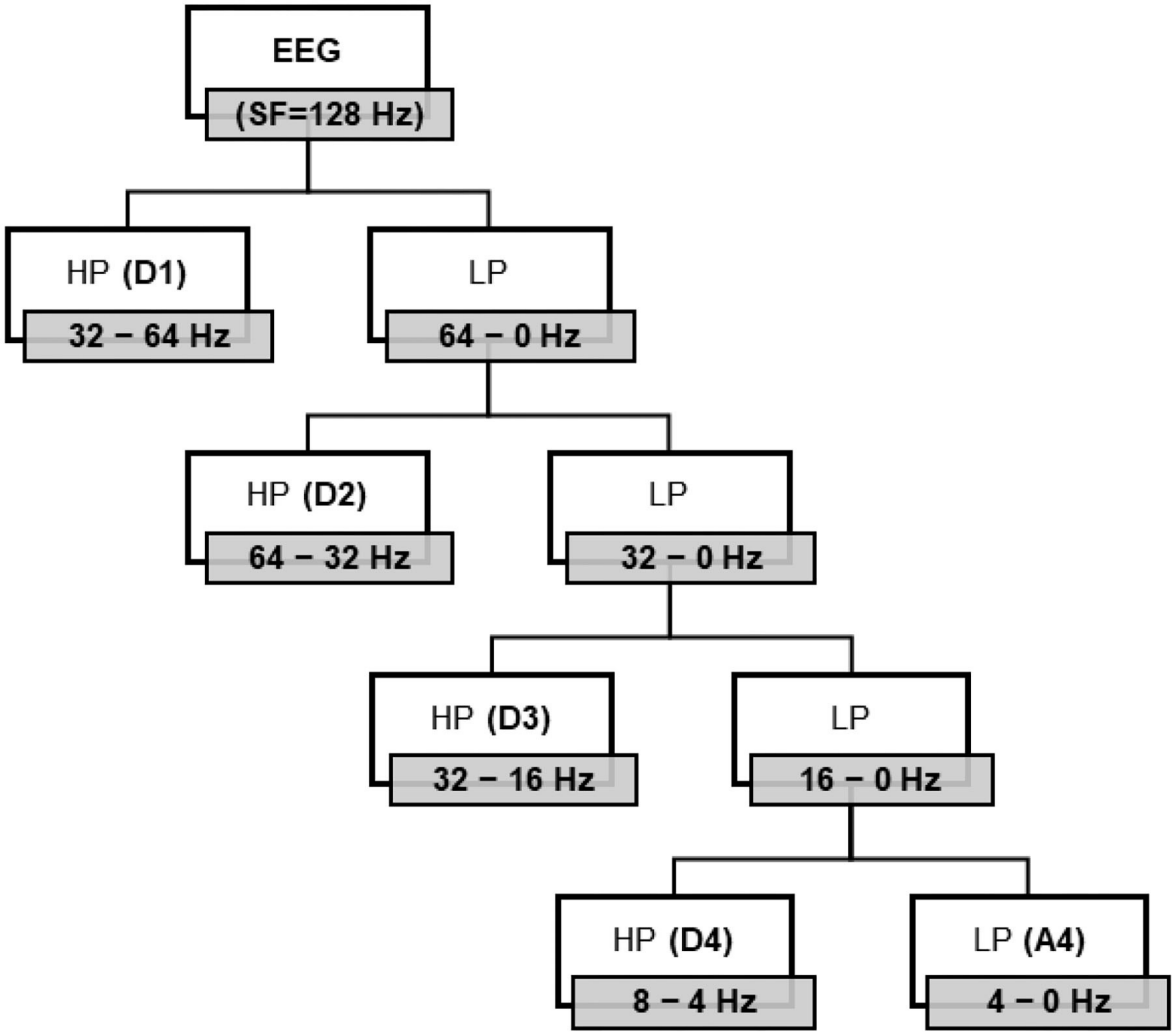
Different levels of DWT decomposition by low-pass (LP) and high-pass (HP) filtering yield A4, D4, D3, D2, and D1 corresponding to delta, theta, alpha, beta, and gamma, respectively, with 128 Hz sampling frequency (SF).

Since the sampling frequency in the present study was 128 Hz, we used four levels of Daubechies (db4) wavelets to decompose EEG signals into five coefficients, namely, A4, D4, D3, D2, and D1. Each coefficient is approximately correlated to the basic frequency bands, namely, (1–4 Hz) delta, (4–8 Hz) theta, (8–13 Hz) alpha, (13–22 Hz) beta, and (22–64 Hz) gamma. The decomposed details D1-D4 and approximation A4 for each of the 14 channels are shown in Figure 3, and their correlated ranges of frequency are listed in Table 3.

**Table 3:** Frequency bands correlated to decomposed coefficients.

**Decomposed coefficient**	**Frequency bands (Hz)**	**Decomposition level**
D1	32–64	Gamma
D2	16–32	Beta
D3	8–16	Alpha
D4	4–8	Theta
A4	0–4	Delta

Moreover, we computed the (Shannon) entropy values as measures of signal complexity and extracted the statistical features that are most commonly used for signals, such as variance, standard deviation, mean, median, 25th and 75th percentile values, root mean square of the average amplitude values, zero and mean crossing rates, and the mean of the signal derivatives. These 10 statistical features and entropy and coefficient values were calculated for the five coefficients for the 14 channels. Thus, the number of DWT features was 12 × 5 × *14 = 840*.

#### 4.3.2. Power Spectral Density

The PSD is an indicator of power in a certain signal in terms of frequency (Xie and Oniga, 2020). PSD is one of the most common feature extraction approaches in neuromarketing research, based on frequency domain analysis. Previous studies (Ohme et al., 2009, 2010; Khushaba et al., 2013) have demonstrated that the PSD obtained from EEG signals is suitable for determining consumer preferences. The PSD approach transforms the data from the time domain to the frequency domain, and vice versa. This conversation is focused on the fast transformation of Fourier, measuring the discrete transformation of Fourier and its opposite. In addition to DWT, we applied the PSD technique in this study to divide each EEG signal into four different frequency bands: theta θ (4–8 Hz), alpha α (8–13 Hz), beta β (13–30 Hz), and gamma γ (30–40 Hz). The MNE package for signal processing was employed for computing PSD and the average power across the frequency ranges.

### 4.4. Calculation of Preference Indices

We implemented various equations to measure the following EEG-based preferences indices (Section 2.2): the AW index, effort index, choice index, and valence. The AW index (frontal alpha asymmetry), measures motivation and desire as higher activation of alpha in the left frontal cortex. We used (Equation 2) stated by Touchette and Lee (2017) to measure the AW scores using electrodes F4 and F3 to find the difference between the right and left PSD divided by their amounts.

(2) $$\begin{array}{l} \text{AW index}=\frac{\alpha \left(F4\right)-\alpha \left(F3\right)}{\alpha \left(F4\right)+\alpha \left(F3\right)} \end{array}$$

The effort index measures effort and cognitive processing as higher theta activation in the prefrontal cortex. We used the following equation to calculate the effort index:

(3) $$\begin{array}{l} \text{Effort Index}=\frac{\theta \left(F4\right)-\theta \left(F3\right)}{\theta \left(F4\right)+\theta \left(F3\right)} \end{array}$$

The choice index measures choice possibility in decision making as higher gamma and beta activation in the frontal cortex (Ramsøy et al., 2018). We used Equation (4) reported by Ramsoy et al. to calculate the choice index for each band individually (gamma and beta) using electrodes AF3 and AF4:

(4) $$\begin{array}{l} \text{Choice index}=\frac{log\left(AF3\right)-log\left(AF4\right)}{log\left(AF3\right)+log\left(AF4\right)} \end{array}$$

The valence measures positive emotion as left frontal activation in alpha and beta bands. We applied different valence equations and investigated the relationships between the self-assessment and different valence measurements. We computed the values of valence using Equations (5), (6), (7), and (8), which are well-explained in literature (Al-Nafjan et al., 2017b).

(5) $$\begin{array}{l} \text{Valence}=\frac{\beta \left(AF3,F3\right)}{\alpha \left(AF3,F3\right)}-\frac{\beta \left(AF4,F4\right)}{\alpha \left(AF4,F4\right)} \end{array}$$

(6) $$\begin{array}{l} \text{Valence}=ln\left[\alpha \left(Fz,AF3,F3\right)\right]-ln\left[\alpha \left(Fz,AF4,F4\right)\right] \end{array}$$

(7) $$\begin{array}{l} \text{Valence}=\alpha \left(F4\right)-\beta \left(F3\right) \end{array}$$

(8) $$\begin{array}{l} \text{Valence}=\frac{\alpha \left(F4\right)}{\beta \left(F4\right)}-\frac{\alpha \left(F3\right)}{\beta \left(F3\right)} \end{array}$$

For all preference indices, we used the PSD to extract frequency band powers from the neuromarketing data because the PSD is based on frequency analysis, unlike DWT, which is based on time and frequency analysis.

### 4.5. Preference Classification Algorithms

In our study, two preference states (“like” or “dislike”) were detected from EEG neuromarketing data. Mainly, we proposed a DNN classifier and compared its performance with those of the KNN, RF, and SVM classifiers. We applied four classifiers, namely, DNN, KNN, RF, and SVM, to discover the optimal preference index and a well-matched classifier with the best accuracy.

RF is an ensemble learning used for classification and regression problems. It consists of a combination of several decision trees where the final outcome class is the mode of all outcome classes of individual trees. Such advantage resulted in low error rates and robustness against over-fitting while preserving computational efficiency (Al-Nafjan et al., 2017b; Teo et al., 2018a). We used the default hyper-parameters of RF in an sklearn package and adjusted the number of trees in the foreset to 500, which all processed in parallel.

#### 4.5.1. DNN Classification

**Figure 4:**
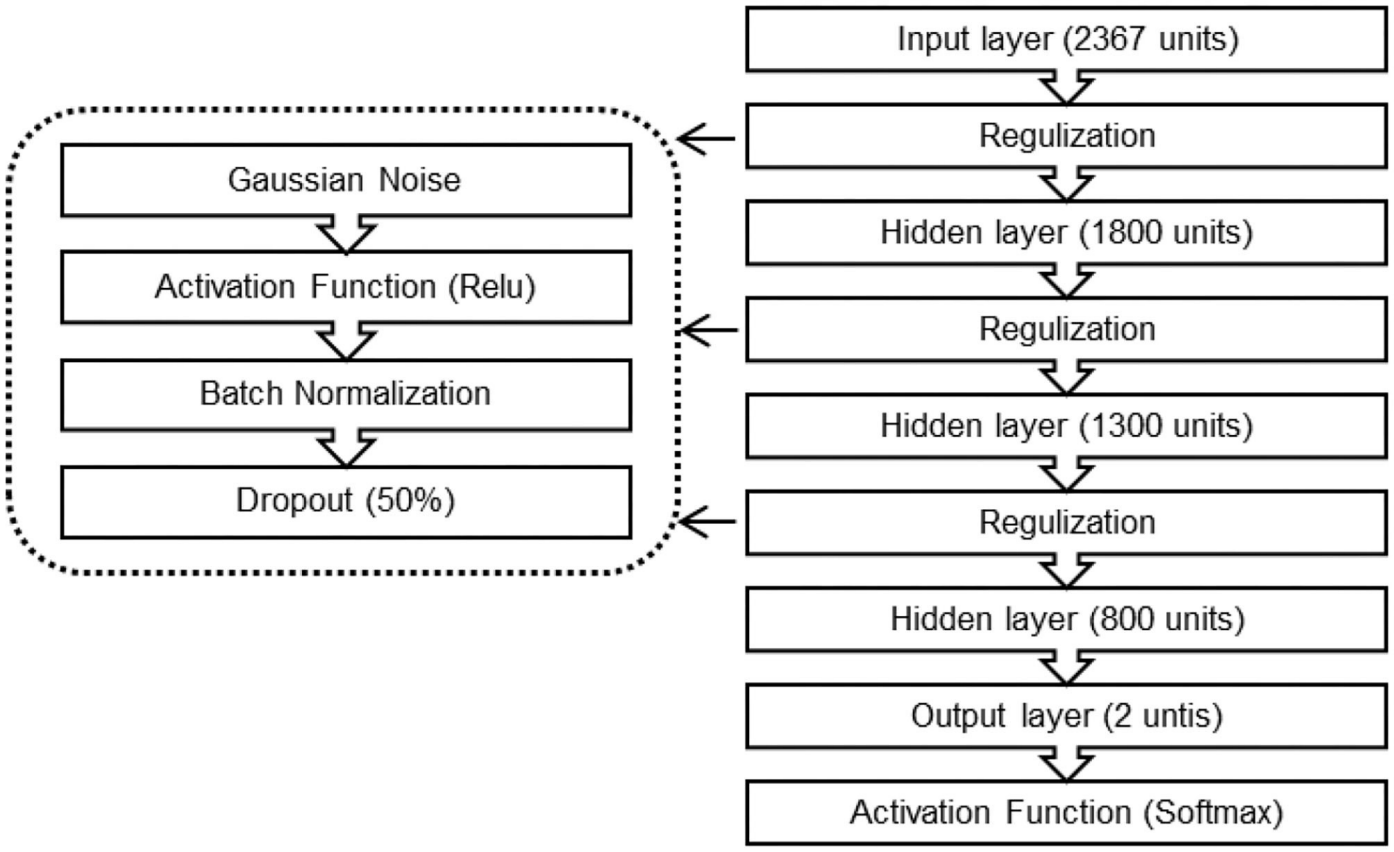
Block diagram of the DNN classifier.

There is an explosive growth of deep learning in machine learning due to its capacity to learn good feature representations from the raw input. DL was able to provide optimal solutions to many problems in natural language processing, image, and speech. With EEG-based BCI, DL has been proven an effective tool to analyze EEG signals (Roy et al., 2019). We aim to investigate the possibility to detect two preference states in the EEG data. We proposed a DNN classifier and compared its performance with the performances of KNN and RF classifiers. The proposed DNN classifier block diagram is shown in Figure 4. The extracted features were first normalized using minimum-maximum normalization (Equation 9) and then fed into the DNN classifier.

(9) $$\begin{array}{l} \begin{array}{c} x_{-}\text{scaled}=\left(x-\min\right)/\left(\max-\min\right) \end{array} \end{array}$$

In our work, we experimented various techniques and architectures. The optimal DNN architecture and properties are as follows:

- Fully connected feed-forward neural network comprised of three hidden layers.
- The input layer consisted of 2,367 units, and each hidden layer consisted of 50% units from its predecessor layer.
- Rectified Linear Unit (ReLu) as activation functions.
- Cross entropy (cost function) to compute the output of the softmax layer.
- The dimension(s) of the output layer was related to the number of target preferences state (2) units.

We used the Adam gradient descent with three objective loss functions: the binary cross-entropy, categorical cross-entropy, and hinge cross functions for training the DNN classifier with the following properties:

- Learning rate was set to 0.001.
- Dropout rate for the input and hidden layers was set to 0.5.
- Stopping criterion, to prevent over-fitting, was determined according to the model performance on a testing set.

Then, we tested our classifier on a test set, which contained approximately 20% of the data samples in the dataset. In our work, we used different approaches to prevent over-fitting including regularization ( such as L1 regularization, L2 regularization, and Gaussian noise), early stopping, and dropout. Adding noise to the DNN model in a relatively small dataset can improve its robustness with regularizing effect and decrease over-fitting.

## 5. Results and Discussion

We detected the preference states (“like” or “dislike”) of the subjects using two different feature extraction methods (PSD and DWT) and four classifiers: DNN, KNN, RF, and SVM. For validation and evaluation, we used various measurements, namely precision, recall, and accuracy. The precision was the percentage of the prediction of “like” states, which was correct. The recall was the percentage of actually expected “like” states. To evaluate the efficiency of the classification algorithms, we split the data into train and test sets with holdout cross-validation.

**Figure 5:**
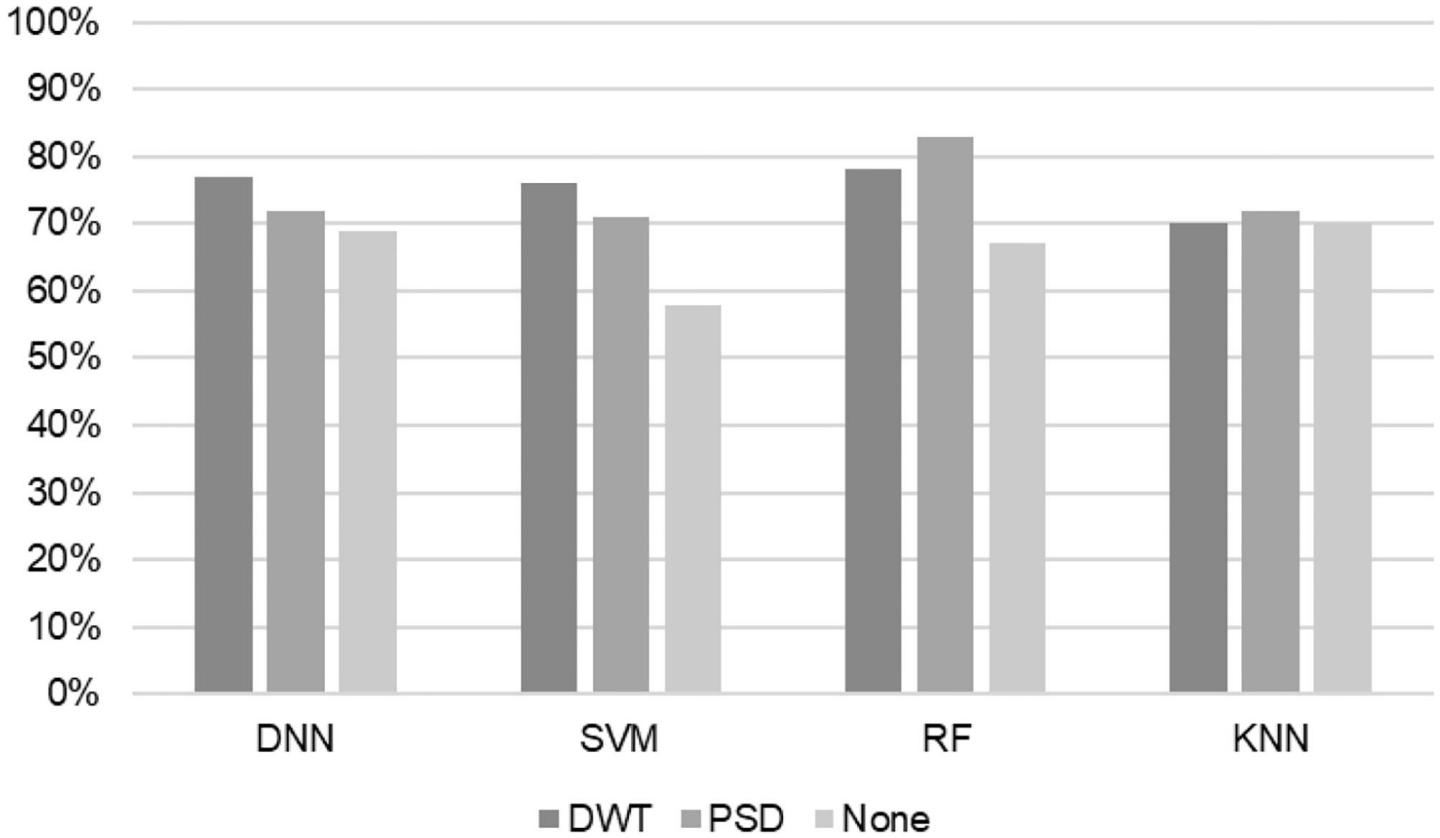
Accuracy results of KNN, RF, SVM, and DNN (hinge activation) using raw EEG signals with and without feature engineering (DWT and PSD).

The proposed DNN classifier was compared with three traditional classifiers for EEG signals: KNN, RF, and SVM using PSD and DWT feature extraction methods as well as various preference indices. Tables 4, 5 list the results of recall, accuracy, and precision results of the KNN, RF, SVM, and DNN algorithms using various activation functions in the DNN: hinge and cross-entropy (categorical and binary) functions. To show the importance of the feature extraction (DWT and PSD), Figure 5 presents the accuracy results of KNN, RF, SVM, and DNN (hinge activation) using raw EEG signals with and without feature engineering (DWT and PSD).

**Table 4:** Classification results of PSD-based feature extraction with/without preference indices and the valence index (V) and different classifiers: KNN, RF, SVM, and DNN using various activation functions in the DNN: hinge, and cross-entropy (categorical and binary) functions.

**Classifiers**	**DNN**									**SVM**			**RF**			**KNN**		
	**Hinge cross**			**Binary cross**			**Categorical cross**											
Preference indices	No (%)	V (%)	All (%)	No (%)	V (%)	All (%)	No (%)	V (%)	All (%)	No (%)	V (%)	All (%)	No (%)	V (%)	All (%)	No (%)	V (%)	All (%)
Accuracy	72	92	93	77	92	93	72	92	92	71	87	86	83	94	93	72	80	78
Recall	72	92	93	77	92	93	72	92	92	71	87	86	83	94	93	72	80	78
Precision	73	92	93	79	92	93	73	92	92	72	88	87	84	94	93	73	80	79

**Table 5:** Classification results of DWT-based feature extraction with/without preference indices and the valence index (V) and different classifiers: KNN, RF, SVM, and DNN using various activation functions in the DNN: hinge, and cross-entropy (categorical and binary) functions.

**Classifiers**	**DNN**									**SVM**			**RF**			**KNN**		
	**Hinge cross**			**Binary cross**			**Categorical cross**											
Preference indices	No	V	All	No	V	All	No	V	All	No	V	All	No	V	All	No	V	All
Accuracy	77	82	83	76	75	80	72	79	80	76	76	81	78	79	87	70	73	73
Recall	77	82	83	76	75	80	72	79	80	76	76	81	78	79	87	70	73	73
Precision	77	82	83	76	75	80	73	79	80	76	76	81	78	79	87	71	73	73

When using PSD-based features, the KNN and SVM classifiers yielded enhanced accuracies of 80 and 87% with the valence index, whereas RF and DNN (binary cross-entropy function) achieved the highest accuracy of 93% with all preference indices. Similar results were achieved with the valence index. Using DWT-based features, the best results were achieved with all preference indices for all classifiers. The KNN and SVM classifiers led to enhanced accuracies of 73 and 81%, respectively. The highest accuracy was 87% with RF and the second-highest accuracy was 83% with DNN and the hinge loss function.

**Figure 6:**
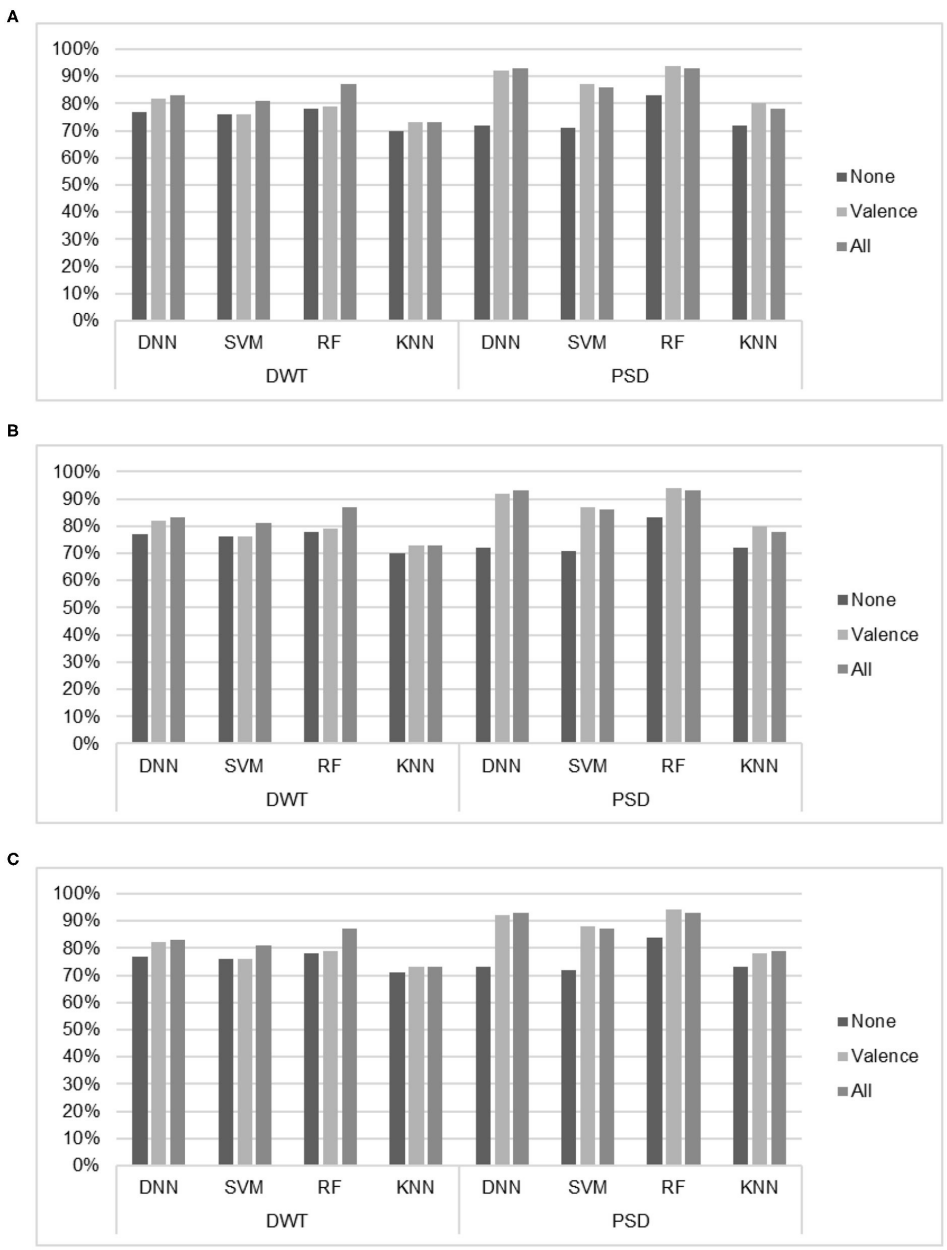
Classification results of the SVM, RF, KNN, and DNN (hinge function) of different combination of preference indices. **(A)** Accuracy. **(B)** Recall. **(C)** Precision.

Figure 6 analyzes the results from the viewpoint of preference indices. We consider the DNN results with hinge loss function as it achieved the best accuracy result compared with other loss functions. About EEG features that exclude preference indices, the best accuracy results reached 83% with RF and DWT-based features.

## 6. Conclusions

A DNN model is proposed for detecting subject preferences from EEG signals using the benchmark neuromarketing dataset. Two kinds of features—PSD and DWT—have been generated from the EEG to obtain a set of 2367 interesting attributes, which demonstrate the EEG task in each experiment. We used various evaluation measures (recall, accuracy, and precision) to test the performance of the classifiers. We built four classifiers, namely, DNN, KNN, RF, and SVM.

The results demonstrated that RF reached the best results in PSD-based and DWT-based features with either valence or all preference indices, however, RF obtained comparable outcomes to DNN. PSD-based features achieved better results in preference detection than DWT-based features. Moreover, combining preference indices leads to better results with either PSD or DWT-based features. This is perhaps the first study that examines in detail the effect of preference indicators in enhancing the performance of classification algorithms.

## Data Availability Statement

Publicly available datasets were analyzed in this study. This data can be found here: https://link.springer.com/article/10.1007/s11042-017-4580-6.

## Author Contributions

MA conceived, designed, and performed the experiment, analyzed and interpreted the data, and drafted the manuscript. MY supervised the analysis and reviewed the manuscript. AA-N co-supervised this study, and contributed to the discussion. All authors have read and approved the submitted version of the manuscript.

## Conflict of Interest

The authors declare that the research was conducted in the absence of any commercial or financial relationships that could be construed as a potential conflict of interest.
